# Diagnostic value of pediatric blood culture bottles for acute postoperative endophthalmitis

**DOI:** 10.6061/clinics/2019/e837

**Published:** 2019-04-04

**Authors:** Tatiana Tanaka, Bruno Fortaleza de Aquino Ferreira, Luiza Manhezi Shin de Oliveira, Juliana Mika Kato, Thais Sabato Romano Di Gioia, Flavia Rossi, Yoshitaka Nakashima, Sergio Luis Gianotti Pimentel, Joyce Hisae Yamamoto, Joao Nobrega de Almeida

**Affiliations:** I Departamento de Oftalmologia (LIM 33), Hospital das Clinicas HCFMUSP, Faculdade de Medicina, Universidade de Sao Paulo, Sao Paulo, SP, BR.; II Divisao Laboratorio Central (LIM 03), Hospital das Clinicas HCFMUSP, Faculdade de Medicina, Universidade de Sao Paulo, Sao Paulo, SP, BR.

**Keywords:** Endophthalmitis, Conventional Culture, Blood Culture Bottle, Postoperative Complications

## Abstract

**OBJECTIVE:**

To report our experience using conventional culture methods (CM) and pediatric blood culture bottles (PBCBs) for vitreous sample culture of acute postoperative endophthalmitis.

**METHODS:**

A retrospective study was conducted at the Department of Ophthalmology, Hospital das Clinicas, HCFMUSP, Faculdade de Medicina, Universidade de Sao Paulo, Sao Paulo, BR, from January 2010 to December 2015, and it included 54 patients with clinically suspected acute postoperative endophthalmitis. Vitreous samples were obtained by vitreous tap or vitrectomy. Samples from January 2010 to December 2011 were cultivated in CM, whereas samples from January 2012 to December 2015 were inoculated in PBCBs. The measured outcome was the yield of positive cultures.

**RESULTS:**

Twenty cases were included in the CM group, and 34 cases were included in the PBCB group. The yield of positive cultures in PBCBs (64.7%) was significantly higher than that in conventional CM (35%, *p*=0.034). *Staphylococcus epidermidis* and *Streptococcus viridans* were the two most commonly found agents.

**CONCLUSION:**

PBCBs can be used successfully in clinically suspected endophthalmitis. The method showed a higher yield of positive cultures than the conventional method. This technique appears to have several advantages over the traditional method: it saves time, as only one medium needs to be inoculated; transportation to a laboratory is easier than in the traditional method, and there is no need to maintain a supply of fresh agar media. The use of PBCBs may be recommended as the primary method for microbiological diagnosis and is especially suitable for office settings and remote clinics.

## INTRODUCTION

Endophthalmitis is a serious intraocular infectious disease associated with elective surgical procedures (75 to 80%), ocular trauma (3.3 to 17%) and endogenous infections (5-15%) (1–3). Suspected cases are initially treated with intravitreal injection of broad-spectrum antibiotics (vancomycin and ceftazidime) (4).

The use of sample cultures is essential to confirming endophthalmitis etiology. Several conditions such as ocular inflammation from noninfectious uveitis, fungal endophthalmitis, and toxic anterior segment syndrome may mimic clinical presentation of endophthalmitis, but bacterial cultures are negative in these cases (5). Identification of the pathogen in cases of endophthalmitis may improve treatment by the early introduction of targeted antibiotics.

Despite advances in molecular assays for detecting pathogens, microbial culture is still the current reference method for the etiological diagnosis of endophthalmitis. Conventional culture methods (CM) use solid or broth media including thioglycolate. However, rates of identification increase when blood culture bottles (BCBs) are used (3,6–9).

The present study aimed to report our own experience using pediatric BCBs (PBCBs) and conventional media for vitreous sample culture in acute postoperative endophthalmitis.

## METHODS

Fifty-four cases of clinically suspected acute postoperative endophthalmitis, attended at the Department of Ophthalmology, Hospital das Clinicas HCFMUSP, Faculdade de Medicina, Universidade de Sao Paulo, Sao Paulo, SP, BR, between January 2010 and December 2015, were retrospectively included. This study was approved by the Institutional Ethics Committee (CAAE: 36514614.4.0000.0068).

Undiluted vitreous samples (200 to 500 mL) were collected by *pars plana* vitrectomy or vitreous tap after local antisepsis, under local anesthesia and before intravitreal administration of antibiotics.

From January 2010 to December 2011, samples were cultivated in CM (thioglycolate) for 5 days at 35°C. From January 2012 to December 2015, samples were inoculated in PBCBs (BACTEC Plus Aerobic/F, BD Diagnostics, USA) and incubated in automated machines for up to 5 days. Positive samples from CM or PBCBs were later inoculated in sheep blood and chocolate agar and incubated for 48 hours under a 5% CO_2_ atmosphere. Identification of causative agents and antibiotic sensitivity tests were performed by VITEK 2 (BioMèrieux, France).

The yields of positive cultures with CM and with PBCBs were compared by using McNemar's test, and the results were considered statistically significant if the *p*-value was less than 5% (*p*<0.05).

## RESULTS

Vitreous samples from 54 patients with endophthalmitis were analyzed. They were associated with phacoemulsification (n=21; 38.9%), trabeculectomy (n=11; 20.4%), extracapsular cataract extraction (n=6; 11.1%), phacoemulsification combined with trabeculectomy (n=5; 9.3%), *pars plana* vitrectomy (n=4; 7.4%), intravitreal bevacizumab injection (n=4; 7.4%), congenital cataract surgery (n=2; 3.7%) and phacoemulsification combined with *pars plana* vitrectomy (n=1; 1.8%).

Thirty-five percent (7 out of 20 cases) of CM and 64.7% (22 out of 34 cases) of PBCB cultures were positive (*p*=0.034) (Table 1). Isolated agents from the 29 positive cultures were *Staphylococcus epidermidis* (n=7; 24.2%), *Streptococcus viridans* (n=6; 20.9%), *Staphylococcus aureus* (n=3; 10.4%), *Haemophilus influenzae* (n=3; 10.4%), coagulase-negative *Staphylococcus* (n=2; 6.9%), *Streptococcus pneumoniae* (n=1; 3.4%), *Enterococcus faecalis* (n=1; 3.4%), *Pseudomonas aeruginosa* (n=1; 3.4%), *Klebsiella oxytoca* (n=1; 3.4%), *Serratia marcescens* (n=1; 3.4%), *Staphylococcus lugdunensis* (n=1; 3.4%), unspecific gram-positive bacilli (n=1; 3.4%) and *Enterobacter cloacae* (n=1; 3.44). Seventy-six percent of the isolates were gram-positive bacteria, mainly *Staphylococcus* spp*.* and *Streptococcus* spp*.* (n=20; 68.9%). Agents isolated from conventional media or in PBCBs according to the associated procedure are described in Table 2.

**Table 1:** Isolated agents from vitreous samples from patients with acute postoperative endophthalmitis using the conventional method (CM) and pediatric blood culture bottles (PBCBs).

Isolated agents	CM (n=7)	PBCB (n=22)	Total
*Staphylococcus epidermidis*	2	5	7 (24.2%)
*Streptococcus viridans*	1	5	6 (20.9%)
*Staphylococcus aureus*	-	3	3 (10.4%)
*Haemophilus influenzae*	-	3	3 (10.4%)
Coagulase-negative *Staphylococcus*	2	-	2 (6.9%)
*Staphylococcus lugdunensis*	-	1	1 (3.4%)
*Streptococcus pneumoniae*	-	1	1 (3.4%)
*Enterococcus faecalis*	-	1	1 (3.4%)
*Enterobacter cloacae*	1	-	1 (3.4%)
*Pseudomonas aeruginosa*	1	-	1 (3.4%)
*Klebsiella oxytoca*	-	1	1 (3.4%)
*Serratia marcescens*	-	1	1 (3.4%)
Unspecific gram-positive bacilli	-	1	1 (3.4%)

**Table 2:** Isolated agents according to procedure and use of conventional media or pediatric blood culture bottles (PBCBs).

Procedure	Conventional media, n (positive cases, %)	Isolated agent (n)	PBCB, n (positive cases, %)	Isolated agent (n)
Phacoemulsification	9 (4, 44.4%)	*Staphylococcus epidermidis* (1)	12 (8, 66.7%)	*Staphylococcus epidermidis*(3)
		Coagulase-negative *Staphylococcus* (1)		*Staphylococcus aureus* (1)
		*Pseudomonas aeruginosa* (1)		*Staphylococcus lugdunensis* (1)
		*Enterobacter cloacae* (1)		*Streptococcus viridans* (2)
				Unspecific gram-positive bacilli (1)
Extracapsular cataract extraction	2 (1, 50.0%)	*Staphylococcus epidermidis* (1)	4 (3, 75.0%)	*Staphylococcus aureus* (1) *Haemophilus influenzae* (1)
				*Klebsiella oxytoca* (1)
*Pars plana* vitrectomy	4 (1, 25.0%)	Coagulase-negative *Staphylococcus* (1)	0 (0)	
Trabeculectomy	3 (0, 0%)		8 (7, 87.5%)	*Staphylococcus epidermidis* (1)
				*Streptococcus viridans* (3)
				*Streptococcus pneumoniae* (1)
				*Enterococcus faecalis* (1)
				*Serratia marcescens* (1)

## DISCUSSION

Endophthalmitis is a rare and devastating complication of ocular surgeries. Rapid identification of the pathogen with adequate treatment may impact visual prognosis. Conventional culture uses solid or broth media; however, PBCBs confer several advantages. Therefore, we demonstrate our experience with using PBCBs for endophthalmitis.

Conventional methods include the use of blood agar, chocolate agar, Sabouraud agar and thioglycolate broth. They require immediate incubation (not available at all ophthalmologic centers), and endophthalmitis positivity varies widely in the literature, ranging from 24 to 72% (3,6–8,10–17). These low sensitivities can be explained by various factors such as the small volume of specimens, the use of antibiotics before the collection of clinical material and the presence of fastidious microorganisms causing endophthalmitis (18).

On the other hand, BCBs confer the possibility of storage at room temperature, microorganism growth with small volume samples, ease of inoculation and low risk of contamination during transport. The use of BCBs is a good alternative in cases of endophthalmitis in areas with limited access to a microbiology laboratory. BCBs also allows the growth of fastidious pathogens (which grow better in atmospheres with high CO_2_ tension) and contain resin that can adsorb antibiotics if the patient has already received them (19). PBCBs have already been accepted as a diagnostic tool for small samples such as blood in pediatric practice, synovial fluid, pleural fluid and peritoneal fluid (19). Kratz et al. have also used PBCBs to test for infectious keratitis and had promising results. Indeed, in some endophthalmitis studies, PBCBs were used (3). Studies using undiluted vitreous samples and BCBs showed average positivity varying from 61% to 100% (3,6–9,19–22). In contrast, Rachitskaya et al. (21) had lower positivity (31.7%) than these values when they used BCBs, likely due to the use of diluted vitreous. Chiquet et al. compared diluted with undiluted vitreous samples using conventional culture methods and suggested that diluted samples were as effective as undiluted samples for microbiological diagnosis of endophthalmitis; however, they also commented that the small number of positive cultures could preclude improving the understanding of the impact of dilution on culture sensitivity (23).

**Figure 1:**
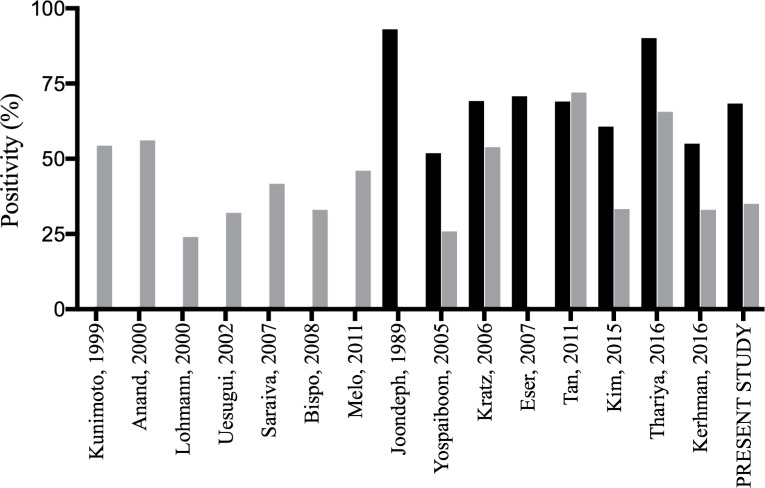
Positivity (%) of vitreous sample cultures of patients with endophthalmitis using the conventional method (gray) and pediatric blood culture bottles (black) in the medical literature and including the present study.

Comparative studies of CM and BCB positivities were carried out in six studies (3,6–9,22); five of them demonstrated a higher positivity with BCBs than with conventional methods (Figure 1). Yospaiboon et al. had a cohort of 27 patients and reported low growth rates overall, 51.9% positivity in BCBs and 25.9% in the traditional method; as discussed by the authors, these results are likely due to the limited volume of samples (0.1-0.2 mL) and previous use of antibiotic therapy (6). Similar to the present study, Kim et al. used PBCBs and CM at different times, reporting positivity of 60.7% and 33.3%, respectively, across 50 samples (8). Thariya et al. presented the largest cohort, with 342 patients, which showed 90.1% positivity in BCBs and 65.6% in CM (9). Only Tan et al. showed a different trend, i.e., a higher positivity with CM culture than with BCBs, although the difference was not statistically significant (7).

In our institution, PBCBs had been used since 2012 for all intraocular samples from patients with endophthalmitis. The present study compared the positivity obtained with the conventional method (previous 2012) and with PBCBs and demonstrated a higher positivity with PBCBs (35% *versus* 64.7%; *p*=0.034). These results are in agreement with previous studies and reinforce the advantages of using PBCBs as an alternative to CM for the etiologic diagnosis of acute postoperative endophthalmitis (3,6,9,10). Figure 1 summarizes the main studies using CM and BCB/PBCBs, including the present study.

The low number of samples for each method and the different periods of inclusion are the main limitations of the present study. Additionally, although the use of PBCBs has several advantages over conventional culture, in cases where anaerobic pathogens are suspected, anaerobic BCBs or broth medium (e.g., thioglycolate broth) should be used (21). Nevertheless, these are the first case series of the advantages of PBCBs produced in Brazil and adding to the international literature. The use of PBCBs should be recommended for microbiological diagnosis of endophthalmitis and is especially suitable for office settings and remote clinics.

## CONCLUSION

PBCBs confer a higher positivity than CM in cultures of vitreous samples of clinically suspected infectious endophthalmitis.
